# Global Status of Phytoplasma Diseases in Vegetable Crops

**DOI:** 10.3389/fmicb.2019.01349

**Published:** 2019-06-27

**Authors:** Shweta Kumari, Krishnan Nagendran, Awadhesh Bahadur Rai, Bijendra Singh, Govind Pratap Rao, Assunta Bertaccini

**Affiliations:** ^1^ICAR-Indian Institute of Vegetable Research, Varanasi, India; ^2^ICAR-Indian Agricultural Research Institute, New Delhi, India; ^3^DISTAL-Phytobacteriology, Alma Mater Studiorum – University of Bologna, Bologna, Italy

**Keywords:** phytoplasmas, vegetables, symptoms, aster yellows, management

## Abstract

The presence of phytoplasmas and their associated diseases is an emerging threat to vegetable production which leads to severe yield losses worldwide. Phytoplasmas are phloem-limited pleomorphic bacteria lacking the cell wall, mainly transmitted through leafhoppers but also by plant propagation materials and seeds. Phytoplasma diseases of vegetable crops are characterized by symptoms such as little leaves, phyllody, flower virescence, big buds, and witches’ brooms. Phytoplasmas enclosed in at least sixteen different ribosomal groups infecting vegetable crops have been reported thus far across the world. The aster yellows phytoplasma group (16SrI) is presently the prevalent, followed by the peanut witches’ broom (16SrII). Wide and overlapping crop and non-crop host ranges of phytoplasmas, polyphagous insect vectors, limited availability of resistance sources and unavailability of environmentally safe chemical control measures lead to an arduous effort in the management of these diseases. The most feasible control of vegetable phytoplasma diseases is a consequence of the development and implementation of integrated disease management programs. The availability of molecular tools for phytoplasma identification at the strain level greatly facilitated this kind of approach. It is moreover essential to understand the molecular basis of phytoplasma-vector interaction, epidemiology and other factors involved in disease development in order to reduce the disease outbreaks. Information on the knowledge about the most widespread phytoplasma diseases in vegetable crops is reviewed here in a comprehensive manner.

## Introduction

Vegetables are short-duration crops, grown during different seasons of the year, which fetch high economic returns. They are excellent sources of nutrients, dietary fibers, phytochemicals and vitamins as well as contribute toward lowering the risk of heart diseases and stroke. Several abiotic and biotic stresses attack vegetables and the phytoplasma-associated diseases represent one of their major constraints in several parts of the world causing significant losses in production yield and quality. Phytoplasmas are obligate prokaryotic wall-less bacteria which multiply in isotonic niches of plant phloem tissues and insect haemolymph. They are pleomorphic, with size variations from 200 to 800 nm and possess a very small genome of about 680–1600 kb. Phytoplasmas are associated with over 600 diverse plant diseases worldwide, mainly transmitted by phloem-feeding insects, especially leafhoppers and plant hoppers (Bertaccini et al., 2014). For several decades the lack of effective methods to identify and characterize phytoplasmas made it not possible to know if the same bacterium was involved in diseases showing similar symptoms on the same or different host plants at various locations. The advent of molecular tools enabled the classification of phytoplasmas into groups and sub-groups, depending in particular on the analysis of 16S rRNA gene sequence (Lee et al., 1998a; IRPCM, 2004). Phytoplasma diseases of vegetables mainly affect plant species belonging to Apiaceae, Asteraceae, Cucurbitaceae, Fabaceae, and Solanaceae. They differ considerably in geographic distribution and number of the various taxonomic groups and subgroups of the associated phytoplasmas. Like other phytoplasma diseases, a number of diseases of vegetable crops are associated with genetically different phytoplasmas which induce similar symptoms in a given plant host. In many instances, economically important diseases of vegetable crops have wild plant species as alternative hosts. Many informative reviews have been published on different aspects of phytoplasma diseases and their significant impact on diverse crops such as ornamental plants, weeds, spices, medicinal plants, and others crops (Bertaccini and Duduk, 2009; Rao et al., 2011, 2017c; Marcone, 2014; Liu et al., 2017; Rao and Kumar, 2017). Abundant information is also available regarding phytoplasma diseases affecting vegetable crops, nevertheless, in a disorganized manner. The objective of this review is to provide a summary of the global status of phytoplasmas infecting vegetable crops.

## Symptomatology

**FIGURE 1:**
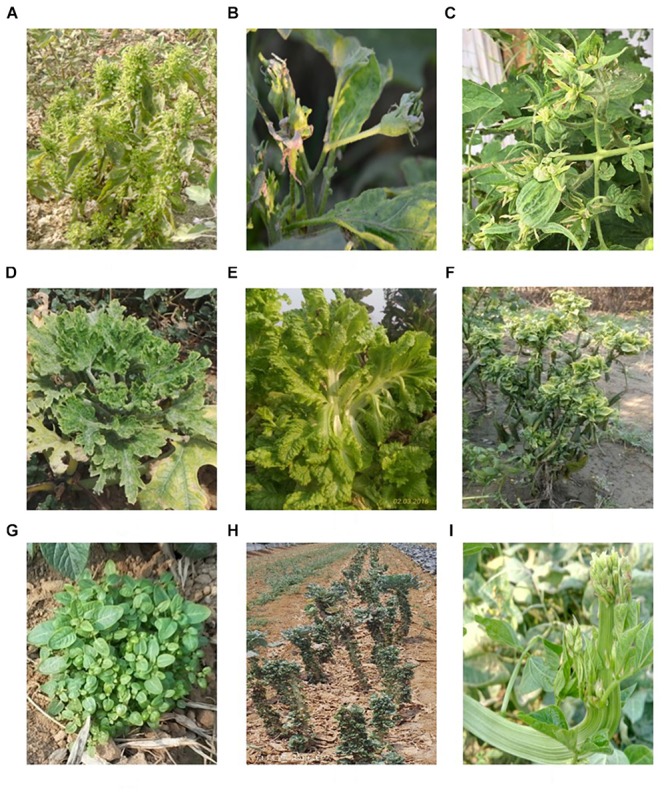
Symptoms in vegetable crops. **(A)** Little leaf in brinjal; **(B)** close up view of brinjal flower showing phyllody; **(C)** big bud in tomato; **(D)** witches broom’ in *Cucurbita pepo;* **(E)** flat stem in lettuce; **(F)** witches’ broom in chili; **(G)** witches’ broom in potato; **(H)** witches’ broom in cabbage; and **(I)** flat stem in cowpea.

The presence of phytoplasmas is associated with a wide range of symptoms including stunting, virescence, shortened internodes, big bud, little leaf, witches’ broom, phyllody, giant calyx, floral malformation, and vascular discoloration (Figure 1). Phytoplasmas may be associated with different symptoms in diverse plant species and/or distinct phytoplasmas may induce similar symptoms in different host species. For instance, phytoplasmas in the 16SrIII group are reported to induce symptoms of little leaf, stunting and witches’ broom in different vegetable crops including cabbage, chili, squash, potato, tomato, and bitter gourd. On the other hand, big bud disease of tomato could be associated with different phytoplasma groups in different geographical areas such as aster yellows (16SrI) in Iran, peanut witches’ broom (16SrII) in China and India, elm yellows (16SrV) in Mauritius, clover proliferation (16SrVI) in United States and “stolbur” (16SrXII) in Russia (Gungoosingh-Bunwaree et al., 2007; Ember et al., 2011; Xu et al., 2013; Sichani et al., 2014; Kumari et al., 2018). Furthermore, several studies reported two or more distinct phytoplasma group infections in a single plant. This is manifested by a stunt disease in broccoli which can be associated with the presence of 16SrI, 16SrIII, and 16SrXII groups of phytoplasmas (Eckstein et al., 2013). Moreover, non-specific symptoms such as yellowing, reddening of leaves, leaf curl, vein clearing, stunting, and fruit malformation may be associated with the phytoplasma presence in vegetable crops (Jung et al., 2003; Pereira et al., 2016).

## Phytoplasma Taxonomy

**FIGURE 2:**
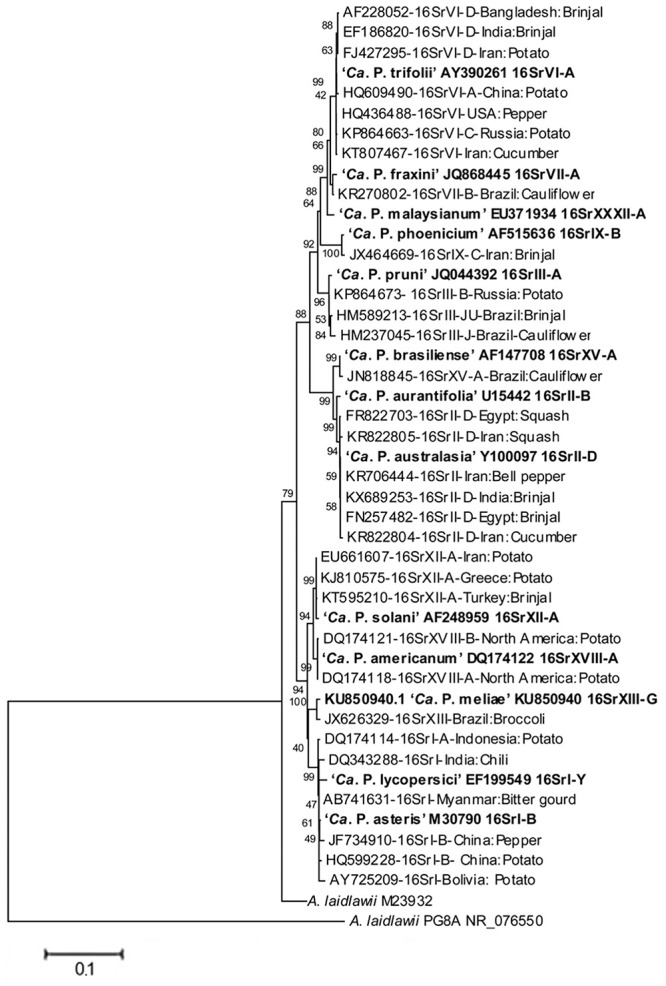
Phylogenetic tree, based on phytoplasma 16S rDNA, showing the relationships among representative of the phytoplasma strains infecting vegetables and related ‘*Candidatus* Phytoplasma’ species (‘*Ca.* P.’). The tree is constructed by neighbor joining method using Mega 6.0 software. GenBank accession numbers are specified in the tree together with ribosomal group or subgroup indication. Numbers on branches are bootstrap values obtained for 1,000 replicates.

These phytopathogenic mollicutes (mycoplasma-like organism – MLO) were named as phytoplasmas in the subcommittee on taxonomy of Mollicutes during 1992 (Lee et al., 2000; IRPCM, 2004). Molecular tools such as PCR/RFLP and nested-PCR on 16S rDNA were developed and established to ascertain a standard and reliable system of identification and classification of phytoplasmas in ribosomal groups and subgroups obtained by RFLP and/or virtual RFLP analyses of the 16S ribosomal gene amplicon or sequence with a number of restriction enzymes (Lee et al., 1998a; Zhao et al., 2009). Since they were only recently cultured (Contaldo et al., 2012, 2016, 2019), biological i.e., classical methods for classification are not available as yet. Currently, phytoplasmas are categorized into 33 ribosomal groups comprising a number of subgroups each (Bertaccini and Lee, 2018). A provisional classification was also established to the taxon ‘*Candidatus* Phytoplasma’ species based on a unique 16S rRNA gene sequence (>1200 bp) and a novel ‘*Ca.* Phytoplasma’ species can be named only if its 16S rRNA gene sequence has <97.5% similarity to that of any of the previously described species or if there are sufficient biological and genetic characteristics to warrant the designation of the new taxon (IRPCM, 2004). The phylogeny of phytoplasma infecting vegetable crops based on phytoplasma 16S rDNA confirms that the two systems of classification are providing the same information (Figure 2) considering that ribosomal groups and subgroups are congruent with the ‘*Candidatus* Phytoplasma’ species defined so far.

## Phytoplasma Detection

In the genomic era, several advanced techniques were employed to detect phytoplasmas therefore properties such as morphology, transmission ability and nucleic acid sequence identity are presently employed for phytoplasma identification and characterization.

### Microscopy

Phytoplasmas were discovered more than 50 years ago by electron microscope observation of ultrathin sections of sieve tubes of shoots and roots in infected plants (Doi et al., 1967). Das and Mitra (2004) visualized the phytoplasmas as bright patches through fluorescence microscopy in the phloem tissue of brinjal, infected with little leaf disease, by staining with fluorochrome 4,6-diamidino-2-phenylindole (DAPI). Microscopy coupled with serology enhances the sensitivity of their detection. In tomato plants infected with “stolbur” and faba bean phyllody, phytoplasmas were confirmed by *in situ* immunofluorescence technique (Cousin et al., 1989). The presence of phytoplasma was also established by electron microscopy in ultrathin sections of sieve tubes of shoots and roots of potato showing witches’ broom (Harrison and Roberts, 1969), as well as in cauliflower and bottle gourd exhibiting symptoms of phyllody and witches’ broom (Chou et al., 1976; Bertaccini et al., 1983) where pleomorphic structures lacking the cell wall were observed. Although microscopy is used as a preliminary detection method of phytoplasmas, it does not allow their identification.

### Grafting and Dodder Transmission

Grafting and dodder transmission helps to transfer phytoplasmas from infected to healthy plants. In crops such as tomato, brinjal, and pepper, phytoplasmas associated with diseases comprising big bud, little leaf, giant calyx, and witches’ broom, were successfully transmitted to healthy plants through grafting (El-Banna et al., 2007; Tohidi et al., 2015). Furthermore, the dodder transmission technique allowed us to confirm the presence of phytoplasmas in cauliflower, tomato and pepper (Choueiri et al., 2007; El-Banna et al., 2007; Rappussi et al., 2012). This is an effective way to study a wide range of phytoplasmas which might provide some insight into their pathogenic properties, such as their host range and cross infectivity, however, it is now not very much used even if it is a very powerful technique to assess phytoplasma ability to infect plants when coupled with their molecular identification.

### DNA Technology

The advancement of nucleic acid based assays such as PCR, nested PCR, PCR-RFLP, and quantitative (q) PCR provides a simple, rapid and reliable means for phytoplasma detection. Since the success of PCR assay primarily depends on an enriched quality of phytoplasma DNA, it has been challenging to detect them in direct PCR assay. This could be attributed to their low-titer and uneven distribution in the host plants. The use of nested-PCR assay increased the accuracy of phytoplasma detection (Bertaccini et al., 2014). Quantitative PCR as a diagnostic tool has also been optimized for detection of several phytoplasmas but it was mainly applied to woody plants such as grapevine and fruit trees since for vegetable crops it is not so relevant considering the short cycle of the infected plants. Besides the conventional PCR assay, new approaches such as loop-mediated isothermal amplification (LAMP) (Hodgetts et al., 2011) digital droplet PCR (Bahar et al., 2018) and next generation sequencing (NGS) (Marcone, 2014) could further enhance pathogen detection in vegetable crops. LAMP has the advantage of low cost, high sensitivity with reduced risk of cross-contamination as it is principally used in the form of a kit which requires less expertise to handle (Liu et al., 2017). However, all these advanced techniques are not often employed for phytoplasma detection in vegetable crops, especially for their short life span. These methodologies could be of great importance for verification of seed transmission reported in several vegetable crops.

## Genetic Diversity

**FIGURE 3:**
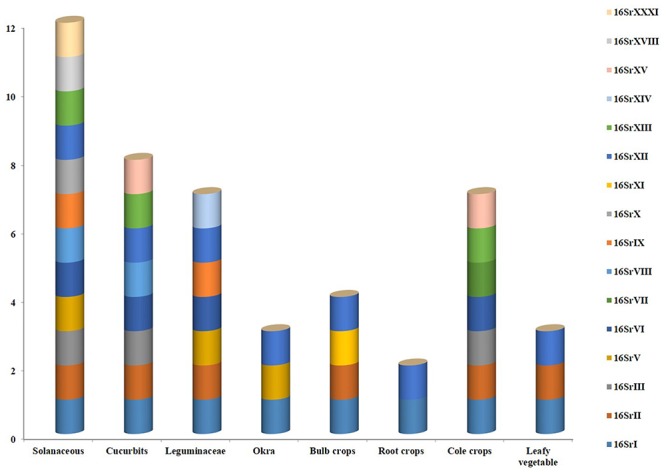
Various phytoplasma groups infecting vegetable crops.

**FIGURE 4:**
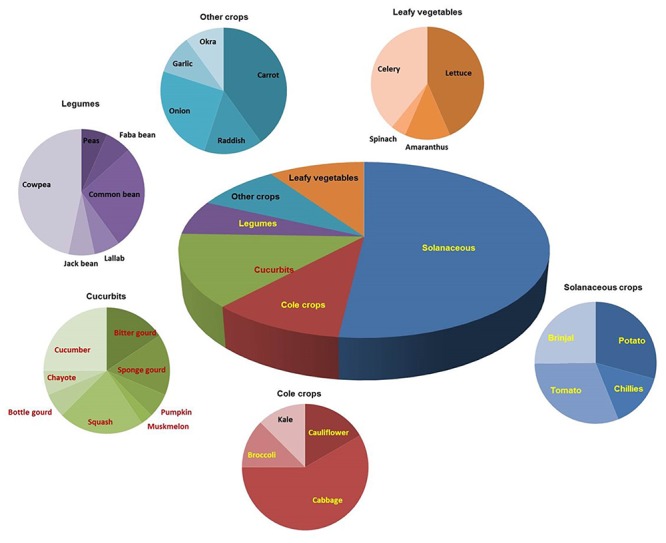
Distribution of phytoplasma diseases in different vegetable crops.

A broad spectrum of genetic diversity is observed among the vegetable-associated phytoplasmas based on their host range and insect vector specificity. Concurrently, sixteen ribosomal groups (16SrI, 16SrII, 16SrIII, 16SrV, 16SrVI, 16SrVII, 16SrVIII, 16SrIX, 16SrX, 16SrXI,16SrXII, 16SrXIII, 16SrXIV, 16SrXV, 16SrXVIII, and 16SrXXXI) and more than twenty one subgroups (16SrI-A, -B, -C, -X; 16SrII-A, -B, -C, -D, -E; 16SrIII-B, -J, -U, -Y; 16SrVI-A, -C, -D, -J; 16SrIX-C; 16SrXII-A, -B; and 16SrXV-A) were reported to be associated with different vegetable species belonging to diverse botanical families including solanaceae, cruciferae, and cucurbitaceae (Table 1 and Figures 3, 4). In the phylogeny based on 16S rDNA nucleotide sequences, phytoplasma ribosomal groups infecting vegetable crops are mainly enclosed into two major clades: the first consists of 4 ribosomal groups (16SrI, 16SrXII, 16SrXIII, and 16SrXVIII) and the second comprises 6 ribosomal groups (16SrII, 16SrIII, 16SrVI, 16SrVII, 16SrIX, and 16SrXXXI) (Figure 2). The phytoplasmas classified among these clades are not grouped based on their geographical distribution nor host plants. This is suggestive of the existence of a wide diversity among phytoplasmas infecting vegetable crops across the globe probably linked to the presence of reservoir plants among the weeds or spontaneous plants in the agricultural environments that represent the main phytoplasma source to the transmission by generalist insect vectors almost never identified.

**TABLE 1:** Distribution of phytoplasma 16Sr groups/subgroups among vegetable crops in the different continents.

**Crop**	**Continents**				
	**Asia**	**America**	**Europe**	**Africa**	**Australia**
**Cole crops**					
Cabbage	16SrI, 16SrVI-D	16SrI, 16SrIII	16SrI		
Broccoli		16SrI, 16SrIII, 16SrXIII	16SrI		
Kale			16SrI, 16SrXII-A		
**Cucurbits**					
Cucumber	16SrII-D, 16SrVI, 16SrXII-A		16SrI-A		
Pumpkin		16SrIII-J			16SrXII-B
Squash	16SrI-B, 16Sr II-D		16SrI	16SrII-D	
Bottle gourd		16SrIII-J			
Sponge gourd	16SrI, 16SrVIII-A	16SrIII-J			
Bitter gourd	16SrI, 16SrII-D				
Muskmelon		16SrIII			
Chayote		16SrI, 16SrIII-J			
**Solanaceous**					
Tomato	16SrI-B, 16SrI-C, 16SrII-A, 16SrII-D, 16SrVI-A	16SrI-A, 16SrI-B, 16SrIII, 16SrVI	16SrI-B, 16SrI-C, 16SrIII, 16SrV, 16SrXII-A	16SrI, 16SrII-A, 16SrII-C, 16SrII-D, 16SrV, 16SrVI, 16SrXII-A	
Brinjal	16SrI-B, 16SrII-A, 16SrII-D, 16SrVI-A,16SrVI-D, 16SrIX-C, 16SrXII-A	16SrIII-J, 16SrIII-U, 16SrXV-A, 16SrVII-B		16SrII-D	
Potato	16SrI-A, 16SrI-B, 16SrII, 16SrIII, 16SrVI-A, 16SrVI-C, 16SrVI-D, 16SrXII-A, 16SrXII-E	16SrI-A, 16SrII, 16SrV, 16SrVI-A, 16SrXIII, 16SrXVIII-A, 16SrXVIII-B	16SrI-B, 16SrX, 16SrXII-A		16SrXII-B
Chili	16SrI-B, 16SrII-D, 16SrVI-D, 16SrXII-A	16SrI-B, 16SrIII, 16SrVI-A, 16SrXXXI	16SrVI, 16SrXII-A	16SrIII	16SrII
**Legumes**					
Pea	16SrII-C, 16SrII-D	16SrI	16SrXII-A		
Faba bean	16SrII		16SrIII-B	16SrII-D	
Garden bean	16SrII-C, 16SrXII-A	16SrI			
Jack bean	16SrII-D	16SrI-B			16SrII-D
Cowpea	16SrI-B, 16SrIX, 16SrXIV-A				16SrV
**Root crops**					
Carrot	16SrII-B, 16SrII-D, 16SrIII, 16SrV, 16SrXII-A	16SrI, 16SrI-B	16SrI-A, 16SrI-B, 16SrII-C, 16SrXII-A		
Radish	16SrI, 16SrII-A, 16SrVI	16SrII	16SrI-B, 16SrXII-A		
Parsnip		16SrI	16SrXII-A		16SrII-E
**Bulb crops**					
Onion	16SrI-B, 16SrII-D, 16SrIX, 16SrXI	16SrI-A	16SrI-A, 16SrI-L	16SrI-B, 16SrXII	
Garlic	16SrIX, 16SrXI	16SrI-A, 16SrIII-J			
**Malvaceae**					
Okra				16SrI, 16SrV, 16SrXII	
**Leafy vegetables**					
*Amaranthus*	16SrII	16SrII			
Spinach	16SrI		16SrXII-A		16SrII-E
Celery	16SrXII-A	16SrI-B	16SrI-B, 16SrI-C, 16SrXII-A		
Lettuce	16SrIX	16SrI-A, 16SrI-B, 16SrIII-J	16SrI-B, 16SrXII-A		

## Geographical Distribution

**FIGURE 5:**
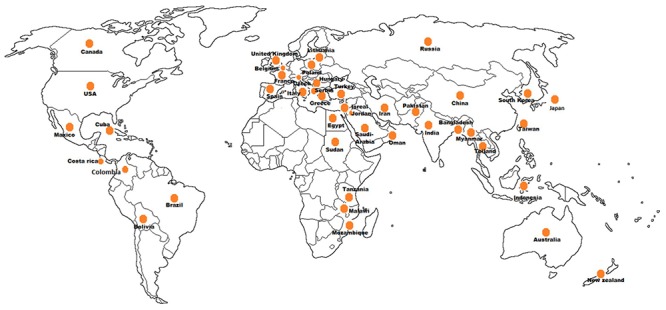
Geographical distribution of phytoplasmas in vegetable crops.

The phytoplasmas infecting vegetable crops are reported in 47 countries distributed among the five continents (Figure 5). The phytoplasmas in the aster yellows group (16SrI) are predominant across different genera followed by the peanut witches’ broom (16SrII), clover proliferation (16SrVI) and “stolbur” (16SrXII-A) groups. Interestingly, among the four subgroups of aster yellows (16SrI-A, -B, -C, -X) infecting vegetable crops, the 16SrI-B subgroup is the most predominant worldwide, whereas the subgroup 16SrI-C seems to be restricted to the Asian countries (Supplementary Table S1).

In the Asian continent phytoplasma strains belonging to 10 ribosomal groups and 16 subgroups have been identified similarly to the detection in the American continent where strains belonging to 12 subgroups in 10 ribosomal groups have been identified. The 16SrIII is the most relevant group of phytoplasmas in the Americas, infecting 10 vegetable crop species after the aster yellows phytoplasma (16SrI), which infects 14 species. The group 16SrIII phytoplasmas appears to be restricted to South America (Argentina, Brazil, Chile, Costa Rica, and Bolivia) except for a report from Mexico, in tomato. Aster yellows and “stolbur” phytoplasmas are well established groups in the European continent where they are emerging as a serious concern for vegetable crops cultivations except for potato, which is infected by “stolbur” (16SrXII-A) phytoplasma. Presence of phytoplasma in vegetable crops has a history of more than 85 years also in Africa and Australia where the tomato big bud disease was first described (Samuel et al., 1933), however, reports of phytoplasma diseases on vegetable crops in Australia are limited; out of the seven phytoplasma groups (16SrI, 16SrII, 16SrIII, 16SrV, 16SrX, 16SrXI, and 16SrXII) reported from Australia, only three (16SrII, 16SrV, and 16SrXII) are infecting vegetable crops. In the African continent three subgroups (16SrII-A, -C, and -D) are ubiquitous, infecting brinjal, tomato, chili, faba bean, and squash (El-Banna et al., 2007; Alfaro-Fernández et al., 2011, 2012; Omar and Foissac, 2012).

## Phytoplasma Diseases in Vegetable Species

### Solanaceous Crops

The solanaceous vegetable species are infected by various groups of phytoplasmas and the most recorded disease is the potato “stolbur” was first observed in Crimea (Russia) in 1935 (Korachevskiy and Semenkova, 1938) and reported during 1950–1960s associated with severe epidemics. So far in potatoes, five phytoplasma groups (16SrI, 16SrII, 16SrIII, 16SrVI, and 16SrXII) and seven subgroups (16SrI-B, 16SrI-C, 16SrII-A, 16SrIII-B, 16SrVI-A, 16SrVI-C, and 16SrXII-A) were identified in Russia (Girsova et al., 2016), while group 16SrVI (clover proliferation) was reported from Korea (Jung et al., 2003). Severe outbreaks are described in Czechia, Hungary, and Romania with yield loss of 30 to 80% (Bogoutdinov et al., 2008; Ember et al., 2011). Other phytoplasma groups (16SrII and 16SrX) were identified in asymptomatic potatoes (Paltrinieri and Bertaccini, 2007). The 16SrXIII and 16SrXVIII groups (‘*Ca.* P. hispanicum’ and ‘*Ca.* P. americanum’) are associated with potato purple top and similar diseases in north America (Lee et al., 2006; Santos-Cervantes et al., 2010) while a 16SrI-F subgroup was detected in Ecuador (Castillo Carrillo et al., 2018) and 16SrXII and 16SrII in New Zealand (Liefting et al., 2009). The big bud disease of tomatoes has different names in various countries and it is typically associated with diverse phytoplasmas. It was reported from western Uttar Pradesh and later detected in many other Indian states (Singh et al., 2012; Kumari et al., 2018) and in northern Australia (Davis et al., 1997). In some European countries this disease is reported as associated with the presence of ribosomal groups including 16SrI, 16SrIII, 16SrV, and 16SrXII (Del Serrone et al., 2001; Vellios and Lioliopoulou, 2007) while in Africa only the latter two groups were identified (Gungoosingh-Bunwaree et al., 2013). The brinjal little leaf disease was first described by Thomas and Krishnaswami (1939) in India with 100% yield loss in epidemics (Rao and Kumar, 2017). Also for this disease, five ribosomal groups were reported: 16SrI (Japan, Bangladesh, and India), 16SrII (Oman and India), 16SrVI (Turkey and India), 16SrIX (Iran) and 16SrXII (Russia and Turkey) (Okuda et al., 1997; Sertkaya et al., 2007; Kelly et al., 2009; Al-Subhi et al., 2011, 2018; Ember et al., 2011; Tohidi et al., 2015; Usta et al., 2015). Similarly phytoplasma infections in chili associated with little leaf, witches’ broom and “brote grande” were reported associated with 16SrVI (India and America) and 16SrII (Egypt) (El-Banna et al., 2007; Randall et al., 2010; Rao et al., 2017a).

### Cruciferous Crops

Crucifer crops are severely damaged by the presence of phytoplasmas that induce phyllody, virescence, witches’ broom and yellowing. The main phytoplasmas detected in cabbage were 16SrVI in Iran and 16SrII in China (Salehi et al., 2007; Cai et al., 2016). The major constraints in cauliflower, cabbage, broccoli, pumpkin, bottle gourd, sponge gourd, bitter gourd, muskmelon, chayote, tomato, and brinjal are also associated with phytoplasmas in Brazil (Montano et al., 2000, 2007) where cauliflower and broccoli stunt were associated with 16SrIII group phytoplasmas. These diseases were characterized by reddening of the leaves, stunting, phloem necrosis and malformed inflorescences (Rappussi et al., 2012; Eckstein et al., 2013). The 16SrXV-A subgroup phytoplasma, predominantly infecting trees, was also reported in Brazil in brinjal and cauliflower crops indicating its wider host range (Canale and Bedendo, 2013). Phyllody and witches’ broom of broccoli, cabbage and kale crops were reported to be associated with 16SrI group phytoplasmas in Europe (Marcone and Ragozzino, 1995; Marcone et al., 1997; Gkavaleka et al., 2012).

### Other Vegetable Crops

Phytoplasma diseases continue to be a concern for tomato, brinjal, potato, cucumber, squash, cabbage, spinach, lettuce, faba bean and chili in Iran where they are associated mainly with 16SrII and 16SrVI phytoplasmas (Zibadoost et al., 2016) but also ribosomal groups 16SrI, 16SrIX, and 16SrXII were reported in various vegetable crops cultivations. 16SrI and 16SrII groups are found to be causing severe yield loss in squash in India and Iran (Hosseini et al., 2011; Salehi et al., 2015; Rao et al., 2017b). Phytoplasma diseases associated with different symptoms in onion and garlic crops were associated with the presence of 16SrI and 16SrIII groups in America and Europe (Vibio et al., 1995; Khadhair et al., 2002; Galdeano et al., 2004; Lee et al., 2011; Mollov et al., 2014) where diverse vegetable crops resulted infected by phytoplasmas in groups 16SrXIII, 16SrXV, 16SrXVIII, and 16SrXXXI. A phytoplasma 16SrXII-A (‘*Ca.* P. solani’) in pea plants cultivated under greenhouse conditions caused more than 25% disease incidence (Zwolińska et al., 2012) and severe losses in carrots (Duduk et al., 2008; Nisbet et al., 2014). The 16SrI and 16SrXII groups were detected in diseased radish (Alfaro-Fernández et al., 2011). Phytoplasmas belonging to sweet potato little leaf (16SrII-D), and ‘*Ca.* P. australiense’ (16SrXII-B) have been reported on cowpea (De La Rue et al., 2001; Saqib et al., 2006). Scattered presence of diseases associated with the presence of groups 16SrII and 16SrXII phytoplasmas in Queensland (Tran-Nguyen et al., 2003; Streteen et al., 2005) and New Zealand (Hill, 1943) was reported.

## Host-Pathogen Interaction

Symptom expressions of phytoplasma infection on vegetable crops are mainly due to alteration in their growth regulating hormones. Genes involved in the expression of growth hormones and floral development were altered upon phytoplasma infection. For instances, genes involved in meristem development (LeWUSCHEL, LeCLAVATA, and LeDEFICIENS) and inflorescence development (FALSIFLORA) altered their expression upon “stolbur” phytoplasma infection in tomato leading to floral abnormalities (Pracros et al., 2006). In case of plant hormones, levels of auxin and cytokinin increased in brinjal plants infected with phytoplasma compared to the healthy (Das and Mitra, 1998). Further, Ding et al. (2013) showed that presence of potato purple top phytoplasma in tomato plants down-regulates the gene encoding a key gibberellic acid (GA) signaling component and a growth repressor known as DELLA protein (gibberrelic acid-insensitive, GAI gene), furthermore, the disruption of gibberellin homeostasis was also observed. Moreover, Buxa et al. (2015) observed profound rearrangement of sieve-element components of phloem tissue such as distortion of sieve-reticulum and changes in the structure of plasma membrane. Due to alterations in gene expression according to the stage of crop during phytoplasma infection, potato plants exhibits an array of symptoms which include purple discoloration or yellowing of upper leaves, apical leafroll, axillary buds and formation of aerial tubers (Longoria-Espinoza et al., 2013).

Some of the mechanisms regulating the phytoplasma-insect-plant host interactions were elucidated after the availability of whole genome sequences and two of those were from phytoplasmas transmitted to *Chrysanthemum* and periwinkle from onion and lettuce, respectively (Oshima et al., 2004; Bai et al., 2006). Some possible pathogenicity factors such as TENGU and SAP11 and/or effector molecules were detected by mining the genomes of OY and AY-WB strains from onion and lettuce and were shown to be related to metabolic and or phenotypic modification identical to those present in the phytoplasma infected plants, such as phyllody and witches’ broom (Bai et al., 2009; Hoshi et al., 2009). Moreover transgenic SAP11-expressing plants down regulated the jasmonic acid synthesis and increased fecundity of insect vectors compared to normal plants (Sugio et al., 2011). A late-flowering phenotype containing a modified expression of flowering-related genes was also observed in transgenic plants expressing SAP11 and correlated its ability to destabilize CIN (CINCINNATA)-TCPs (Chang et al., 2018). A genomic region containing four glycolytic genes was duplicated in the OY-W genome was a unique gene structure not identified in any other bacterial, considering that the ATP synthesis in phytoplasmas might be dependent on the glycolytic pathway (Oshima et al., 2004) the higher consumption of the carbon source may cause more severe symptoms in the OY-W-infected plant (Oshima et al., 2007). It would be essential to study host-phytoplasma interactions and their relationship with alteration in growth hormones and other metabolic pathways. Treatment of plants with anti-growth regulatory compounds (anti-auxin, anti-gibberllins, anti-cytokinin, etc.) or other pathway-focused molecules, according to their interactions may provide an insight into improved phytoplasma disease management.

## Phytoplasma – Virus Mixed Infection

Symptoms of phytoplasma infection on vegetable crops are very often confused with those caused by viruses. Most of the symptoms such as yellowing, witches’ broom, stunting and malformation of foliage could be associated with the presence of both phytoplasma and virus. Several reports of virus and phytoplasma co-infections in vegetable crops are available. Mixed infection of pepper by phytoplasmas belonging to 16SrIII group with two begomoviruses, *Tomato yellow leaf curl virus* (TYLCV) and *Tomato Chino la Paz virus* (ToChLPV), were reported in Mexico. The diseased plant exhibited typical symptoms of leaf chlorosis, necrotic lesion on leaves, shortened internodes, discoloration, distortion, and thickening of leaves (Lebsky et al., 2011). Swarnalatha and Krishna Reddy (2014) observed mixed infections of *Tomato leaf curl New Delhi virus* and big bud phytoplasmas in tomato crops in different regions of Karnataka, India with disease incidence ranging from 1.2 to 7.2%. Moreover, mixed infection in brinjal by phytoplasmas belonging to the 16SrVI group along with begomoviruses was reported in Meerut, India (Singh et al., 2015). Mixed infections of *Potato virus X*, *Potato virus Y* and little leaf phytoplasmas was also reported in brinjal plants showing little leaf and mosaic mottling disease in India (Kumar et al., 2016). The occurrence of mixed infection may enhance disease severity and yield loss when compared to those of a single infection. Hence, synergistic interactions between phytoplasmas and viruses should be studied in detail especially in vegetable crops.

## Epidemiology

Phytoplasmas infecting vegetable crops are evolving into alarming complexities worldwide. Potato purple top, tomato big bud, and brinjal little leaf are among the most widespread diseases causing extensive damages. Phytoplasma infection led to significant yield losses in brinjal (40%), tomato (60%), pepper (93%), potato (30–80%), and cucumber (100%) in different parts of the world (Bogoutdinov et al., 2008; Navratil et al., 2009; Rao and Kumar, 2017). Insect vectors play a key role in the epidemiology of these phytoplasma diseases. Known insect vectors are leafhoppers, plant hoppers and psyllids enclosed in three major groups: Cicadellidae, Fulgoromorpha, and Psyllidae. However, a large number of insect vectors responsible for diseases transmission belong to the Cicadellidae family. Among vegetable crops, aster yellows phytoplasma is transmitted by *Macrosteles quadrilineatus* (Zheng-Nan et al., 2013), while peanut witches’ broom phytoplasma infecting Cucurbitaceae (cucumber, squash), Solanaceae (pepper), and Cruciferae (radish) is transmitted by *Orosius albicinctus*, *Macrosteles laevis*, and *Orosius argentatus* (Tran-Nguyen et al., 2003; Salehi et al., 2015). Clover proliferation phytoplasma (16SrVI), responsible for severe yield loss in brinjal and cabbage, is transmitted through *Empoasca devastans*, *Hishimonus phycitis*, and *Circulifer haematoceps* (Thomas and Krishnaswami, 1939; Salehi et al., 2007). The reported vectors of “stolbur” phytoplasma (16SrXII-A) in vegetable crops are *Macrosteles laevis*, *Hyalesthes obsoletus*, and *Circulifer tenellus*. In particular, *C. tenellus* has been identified as the potential vector of potato purple top disease. A single insect-vector species may transmit one or more phytoplasmas and in contrast a single phytoplasma may be transmitted by different insect species (Lee et al., 1998b). Moreover, several weed species are reported which act as an alternate host for phytoplasmas. For instance, prominent weeds identified as potential reservoir hosts are *Convolvulus arvensis*, *Cirsium arvense*, *Cuscuta* sp., *Urtica dioica*, and *Euphorbia falcata* for “stolbur” phytoplasma (16SrXII-A); *Datura stramonium*, *Cannabis sativa*, *Portulaca oleracea*, and *P. grandiflora* for brinjal little leaf phytoplasma (16SrVI) in the Asian and European continents (Navratil et al., 2009; Ember et al., 2011; Rao and Kumar, 2017).

There are also reports about the seed transmission in some vegetable crops such as winter oil seed rape and tomato (Calari et al., 2011), and *Brassica napus* (Satta et al., 2019) of phytoplasmas belonging mainly to the 16SrI-B group at the seedling stage. This is possible especially when the phytoplasma infection is transmitted to the late growing stages of the crops since it is not modifying the seed production. However, in the majority of these seedlings after the fourth leaf stage the presence of phytoplasmas is greatly reduced (Olivier et al., 2010; Satta et al., 2019). However, considering that phytoplasma presence is not verified by any quarantine protocol nor by seed producers, the movement of seeds from infected plants could imply the geographic dissemination of the pathogen and therefore the associated diseases in still uncontaminated areas should be further studied.

It is clear that adjacent non-crop susceptible species may be responsible for maintenance and perpetuation of the inoculum, through disease transmission to subsequent crops or other economically important crop species. In most studies, potential insect vectors were identified through PCR assay by phytoplasma detection in the insect body. Upon feeding the infected plant sap, all insects acquiring phytoplasmas may not be able to transmit them, hence, it is essential that the insect vector is confirmed through transmission studies. Phytoplasma identification in vegetable crops is based on characterization of their 16S rDNA region through PCR-RFLP assay and/or sequencing. Assessment of yield loss associated with the presence of phytoplasma diseases and the role of weather and environmental parameters in their transmission are yet to be determined on vegetable crops.

## Management

Several approaches were suggested for the management of phytoplasma diseases and their insect vectors. Unfortunately, not even a single effective control measure has been identified to date. Control of insect vectors through pesticides is a plausible way to limit the spreading of phytoplasma diseases infecting vegetable crops. However, complete elimination of vectors is not achievable despite heavy dosage application of chemicals (Firrao et al., 2007). The severity of brinjal little leaf disease was reduced by rouging symptomatic plants and spraying insecticides (Sohi et al., 1974). Treatment of diseased plants using tetracycline is another method of management of phytoplasma diseases. However, this is not a long-term prophylactic method since such plants, when exposed to insect vectors again, are prone to re-infections. Furthermore, antibiotics are too costly and their application is prohibited in several countries. The effect of tetracycline was reported to be only a temporary remission of symptoms in brinjal infected with little leaf disease and could not eliminate the pathogen completely in the host plant (Raychaudhuri et al., 1970). Moreover, flowers and fruits were not observed in any of the brinjal cultivars treated with antibiotics (Upadhyay, 2016). Varma et al. (1975) demonstrated that spraying gibberellic acid on infected brinjal plants induced symptom recovery and an increasing recovery rate was reported with gibberellic acid treatment followed by ledermycin. Several bio-agents are also utilized for plant disease management. Treatment of tomato plants infected with “stolbur” phytoplasma with arbuscular mycorrhizal (AM) fungi led to reduced symptom expression and degeneration of phytoplasma cells (Lingua et al., 2002). The effects of different molecules such as biophenicol, chloramphenicol, enteromycelin, lycercelin, paraxin, roscillin, camphicillin, oxytetracycline, chlorotetracycline, and rose/clove/eucalyptus oils on brinjal cultivars infected with phytoplasmas were studied and found to be quite ineffective since they did not display any significant disease control. Treatment of plants with a host defense inducer could be another prospective disease control mechanism. Sanchez-Rojo et al. (2011) studied the effect of salicylic acid on potato plant infected with the purple top disease and found a significant reduction in their symptom expression. Wu et al. (2012) showed that pre-treatment with two applications of salicylic acid 2 and 4 days before phytoplasma inoculation by insects significantly reduced purple top disease symptoms in tomato plants. Pre-treatment of salicylic acid also caused the upregulation of the expression of three defense-related genes which included LeWRKY1, LeMPK3 and LePRP1, 3 days after phytoplasma inoculation.

**FIGURE 6:**
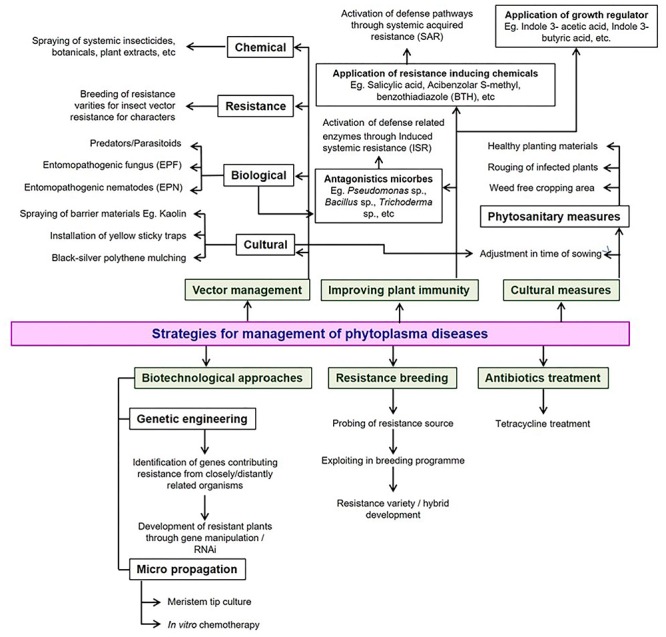
Possible strategies available for the management of phytoplasma diseases in vegetable crops.

Developing cultivars resistant to either phytoplasmas or their insect vectors would be a long-lasting tool for the control of phytoplasma diseases. Limited work was accomplished on the development of resistant genotypes of vegetable crops. Chakrabarti and Choudhury (1975) reported that the wild relatives of brinjal, *Solanum integrifolium* and *S. gilo* were resistant to the little leaf disease. Other management strategies such as rouging of infected plants, adjustment of date in sowing, use of clean propagating material, rotation with non-host crops, and removal of weeds coupled with vector control are effective methods for the containment of phytoplasma-associated diseases. The dependency of the phytoplasmas on a living host for their survival makes it impossible their management with a single chemical and is quite different from the management carried out for fungi or bacteria. Hence, an integrated approach may be the most viable and sustainable option by integrating components of cultural, physical, biological, resistance and chemical applications (Figure 6).

## Conclusion

The geographical distribution and impact of phytoplasma diseases primarily depends on the host range as well as the feeding preference of insect vectors. One plant species can be infected by a single or multiple phytoplasmas and an individual phytoplasma strain may infect numerous plant species indicating the frequent lack of host-specificity of phytoplasmas. Furthermore, intermittent detection of phytoplasmas in new crops or new regions indicates continuous spread of the vector which represents a threat to new crops and new horizons. In addition to phytoplasma diagnostics, future research priorities should be focused on vector-phytoplasma interactions; vector biology; role of weather parameters in disease epidemics; development of resistant varieties; and crop and region specific integrated disease management modules. Priorities for future research should be based on mechanisms of spread of the vector(s), verification of seed transmission and development of resistant varieties to control phytoplasma-associated diseases.

## Author Contributions

SK and KN outlined and conceived the review. SK, KN, GR, and AB prepared the draft of the manuscript. GR, AB, AR, and BS corrected and reviewed the manuscript.

## Conflict of Interest Statement

The authors declare that the research was conducted in the absence of any commercial or financial relationships that could be construed as a potential conflict of interest.
