# Supplementary material for: Global Status of Phytoplasma Diseases in Vegetable Crops
Source: Front Microbiol. 2019 Jun 27;10:1349. doi: 10.3389/fmicb.2019.01349 (PMC6610314; doi:10.3389/fmicb.2019.01349)
Supplement: Supplementary file 1 [file Table_1.DOCX]

**Table S1.** Phytoplasmas detected in vegetable crops worldwide.

| **Symptoms** | **Country** | **GenBank Accession Number** | **Ribosomal group/ subgroup** | **References** |
| --- | --- | --- | --- | --- |

| **Cole Crops (Cruciferae)**  ***Brassica oleracea*var. *capitata* (cabbage)** |
| --- |

| Stunt | Brazil | JQ766307 | | 16SrIII | | Rappussi et al. 2012, Eur. J. Pl.Pathol. 133, 829–840. | | | | | |
| --- | --- | --- | --- | --- | --- | --- | --- | --- | --- | --- | --- |
| Witches’ broom | China | - | | 16SrI-B | | [Mou](http://apsjournals.apsnet.org/author/Mou%2C+Hai-qing) et al. 2012, J.Phytopath.160,304-307. | | | | | |
| Stunt | Greece | HE601634 | | 16SrI-B | | Gkavaleka et al. 2012, J. Pl.Pathol. 94, S4.85-S4.105. | | | | | |
| Short thick leaf | Hungary | - | | 16SrI | | Fodor et al. 1999, ActaPhytopatht. Entom. Hung. 34, 1-6. | | | | | |
| Iranian cabbage yellows | Iran | DQ195214 | | 16SrVI | | Salehi et al. 2007, Pl.Pathol.56,669–676. | | | | | |
| Witches’ broom, phyllody, big bud |  | - | | 16SrVI-A | | Zibadoost et al. 2016, Aus. Pl. Dis. Notes 11, 3 | | | | | |
| .Witches’ broom, stunting | India | KX6971553 | | 16SrVI-D | | Khasa et al. 2017, Physiol. Mol. Biol. Pl. 24, 203-210. | | | | | |
| Virescence, phyllody | Italy | - | | 16SrI | | Marcone and Ragozzino 1995, J.Pl.Dis.Prot. 102, 449-460 | | | | | |
| Stunt, death of flower bud | Poland | - | | 16SrI | | Kaminska and Berniak 2012, J. Agric. Sci. 4, 219-226. | | | | | |
| Phyllody | Serbia | - | | 16SrI-B | | Duduk et al. 2007, Bull.Insectol.60, 341–342. | | | | | |
| Koolsard | United Kingdom | - | | 16SrI-B | | Keane et al. 1996, Diagn. Crop Prod.65, 263-268. | | | | | |
| Purpling | USA | - | | 16SrI-B | | Lee et al. 2004, Int. J. Syst.Evol.Microbiol. 54, 1037–1048. | | | | | |
| ***Brassica oleracea*var. *italica* (broccoli)** | | | | | | | | | | | |
| Stunt | Brazil | JX626329 | 16SrI, -III, -XIII | | | | Eckstein et al. 2013, J. Phytopath. 161, 442–444. | | | | |
| Phyllody | Italy | - | 16SrI-B | | | | Marconeand Ragozzino 1995, J. Pl. Dis. Prot. 102, 449-460. | | | | |
| ***Brassica oleracea*var. *palmifolia*(kale)** | | | | | | | | | | | |
| Phyllody | Italy | - | | | 16SrI-B | | | Marcone and Ragozzino 1995, J.Pl.Dis.Prot. 102, 449-460. | | | |
| Reddening, stunt | Serbia | - | | | 16SrXII-A | | | Trkulja et al. 2011, Bull.Insect.64(Suppl), S81-S82. | | | |
| ***Cucurbitaceae***  *Cucumissativus* (cucumber) | | | | | | | | | | | |
| Phyllody | Iran | KR822804 | | | 16SrII-D | | | | Salehi et al. 2015, Bull. Insect.68, 311-319. | | |
|  |  | JN574839 | | | 16SrII | | | | Dehghan et al. 2014, J. Pl.Pathol. 50, 185-186. | | |
|  |  | - | | |  |  |  |  | Tazehkand et al. 2010, Ir. J.Phytopath. 158, 713-720. | | |
|  |  | KT807467 | | | 16SrVI | | | | Zibadoost et al. 2016, Aus. Pl. Dis. Notes 11, 3. | | |
| Phyllody, virescence |  | KR633068 | | | 16SrVI | | | | Esmailez-Hosseini et al. 2015, New Dis. Rep. 32, 5. | | |
| Yellows, short internodes | Lithuania | KX298483 | | | 16SrI-A | | | | Valiunas et al. 2017, Pl. Dis.101, 379-379. | | |
| Witches’ broom | Turkey | KX977570  KR080212 | | | 16SrVI-A  16SrXII-A | | | | Usta et al. 2017, Yyü Tar.Bil.Derg. 27, 299-308. | | |
| ***Cucurbitamoschata*(pumpkin)** | | | | | | | | | | | |
| Yellow leaf curl | Australia | - | | | 16SrXII-B | | | | | | Streteen et al. 2005, Aus. Pl.Pathol.34, 103–105. |
| Little leaf, fruit malformation | Brazil | - | | | 16SrIII-J | | | | | | Montano et al. 2006, J. Pl.Pathol. 88, 225-229. |
| ***Cucurbitapepo* (squash)** | | | | | | | | | | | |
| Little leaf, witches’ broom | Egypt | FR822703- FR822705 | | | 16SrII-D | | | | | | Omar and Foissac 2012, Eur. J. Pl. Path. 133, 353–360. |
| Witches’ broom | India | KY457228 KY471168 | | | 16SrI-B | | | | | | Rao et al. 2017, New Dis. Rep. 35, 33. |
| Phyllody | Iran | KR822805 | | | 16SrII-D | | | | | | Salehi et al. 2015, Bull. Insect.68, 311-319. |
| Yellowing | Italy | - | | | 16SrI | | | | | | Minucci et al. 1995, Inf.toreFitopatol.45, 61-64. |
| Yellowing | Oman | - | | | 16SrII-D | | | | | | Al-Subhi et al. 2018, Pl.Dis. 95, 360. |
| ***Lagenariasiceraria*(bottle gourd)** | | | | | | | | | | | |
| Little leaf, yellows | Brazil | - | | | 16SrIII-Y | | | | | Montano et al. 2015, Phytopath. Moll. 5(1-Suppl.), S81-S82. | |
| Virescence, phyllody | India | LT594117 LT594118 | | | 16SrI-X | | | | | Tripathi et al. 2017, Pl.Dis.101, 1949. | |
| ***Luffacylindrica*(Indian luffa*)*** | | | | | | | | | | | |
| Little leaf yellowing | Brazil | - | | | 16SrIII-Y | | | | | Montano et al. 2015, Phytopath. Moll. 5(1-Suppl), S81-S82. | |
| Witches’ broom |  | - | | | 16SrIII | | | | | Montano et al. 2007, Bull. Insect. 60, 277-278. | |
| Little leaf | India | HM467913 | | | 16SrI | | | | | Kumar *et al*. 2010, Aus. Pl. Dis. Notes 5, 117-119. | |
| Witches’ broom | Taiwan | - | | | 16SrVIII-A | | | | | Lee et al. 1993, Phytopathol.83, 834-842. | |
| ***Momordicacharantia* (bitter gourd)** | | | | | | | | | | | |
| Little leaf | India | KX179474 | | | 16SrI | | | | | Venkataravanappa et al. 2017, Arch. Biol. Sci.69, 707-714. | |
| Little leaf | Myanmar | AB741631 | | | 16SrI | | | | | Win et al. 2014, Trop. Pl.Pathol.39, 82-88. | |
| Witches’ broom | Brazil | - | | | - | | | | | Jimenez Nilda and Montano 2010, Trop. Pl.Pathol. 35, 381-384. | |
| Phyllody | Thailand |  | | | 16SrI | | | | | Sdoodee et al. 1999, J.Bioch., Mol. Biol.Bioph. 3, 133-140. | |
| ***Sicanaodorifera* (musk melon)** | | | | | | | | | | | |
| Witches’ broom | Brazil | - | | | 16SrIII | | | | | Montano et al. 2007, Bull. Insect. 60, 287-288. | |
| ***Sechiumedule* (chayote)** | | | | | | | | | | | |
| Witches’ broom | Brazil | AF147706 AF147707 | | | 16SrIII-J | | | | | Montano et al. 2000, Pl.Dis. 84, 429-436. | |
|  | Costa Rica | - | | | 16SrI | | | | | Villaloboset al. 2002, Pl.Dis. 86, 330. | |

| **Solanaceae**  ***Solanumlycopersicum*(tomato)** | | | | | | | | | | | |
| --- | --- | --- | --- | --- | --- | --- | --- | --- | --- | --- | --- |
| “Hoja de perejil” | Bolivia | | | | EF199549 | 16SrI | | | Arocha et al. 2007, Int. J. Syst.Evol.Microbiol. 57, 1704-1710. | | |
| Big bud | Brazil | | | | - | 16SrIII | | | Amaral-Mello et al. 2006, Int.J. Pest Manag.52, 233-237. | | |
| Big bud | China | | | | - | 16SrVI | | | Du et al. 2013, J.Phytopath. 161, 870–873. | | |
|  |  |  |  |  | - | 16SrII | | | Xu et al. 2013, J.Phytopath. 161, 430-433. | | |
| Stunting, shoot proliferation |  |  |  |  | JX162603–JX162606 | 16SrII-A | | | Dong et al. 2013, J. Gen. Pl.Pathol. 79, 366-369. | | |
| Short internodes stunting | Cuba | | | | JN383913 | 16SrI | | | Zamora et al. 2014, New Dis.Rep. 30, 10 | | |
| Big Bud | Egypt | | | | FR822700 FR822698 | 16SrII-D 16SrII-A | | | Omar and Foissac 2012, Eur. J. Pl. Path. 133, 353–360. | | |
|  |  |  |  |  | EU232714 | 16SrII | | | **El-Banna** et al. 2007, Egypt. J.Virol. 4, 93-111. | | |
| Hypertrophic calix | France | | | | - | 16SrXII-A | | | Pracros et al. 2006, Mol. Plant-Microbe Inter. 19, 62–68. | | |
| Big bud | Greece | | | | - | 16SrI  16SrXII | | | Vellios and Lioliopoulou 2007, Bull. Insect.60, 157-158. | | |
| Big bud | India | | | | KF975588 | 16SrII | | | Swarnalatha and Krishna Reddy 2014, Pest Man. Hort.Ecosyst. 20, 59-68. | | |
| Witches’ broom | India | | | | JX104335 | 16SrII-D | | | Singh et al. 2012, Phytopath. Moll. 2, 69-71. | | |
| Big bud, little leaf | Iran | | | | KT807466 | 16SrVI-A | | | Zibadoost et al. 2016, Aus. Pl. Dis. Notes 11, 3. | | |
|  |  |  |  |  | GU797557 | 16SrI-B | | | Sichani et al. 2014, J.Pl.Prot. Res. 54, 1-8. | | |
| Witches’ broom |  |  |  |  | KC182528 | 16SrII-D | | | Salehi et al. 2014, Ir.J. Crop Prot. 3, 377-388. | | |
| Shoot proliferation, yellowing, |  |  |  |  | - | 16SrVI, 16SrI | | | Moslemkhani et al. 2014, J. Crop Prot. 3, 573-580. | | |
| Hypertrophic calyx | Italy | | | | - | 16SrI, -III, -V, –XII | | | Del Serrone et al. 2001, Phytopath.Medit.40, 137-142. | | |
| Yellowing | Japan | | | | - | 16SrI | | | Okuda et al. 1997, Pl.Dis. 81, 301–305. | | |
| Calixhypertrophy | Jordan | | | | - | 16SrVI | | | Anfoka et al. 2003, J.Phytopathol. 151, 223-227. | | |
| Witches’ broom,stunting | Mauritius | | | | - | 16SrXII, 16SrI, 16SrV | | | Gungoosingh-Bunwaree et al. 2013, [Aus. Pl.Pathol](http://link.springer.com/journal/13313).42, 659–665. | | |
| Short internodes stunting |  |  |  |  | - | 16SrI-C, 16SrV | | | Gungoosingh-Bunwaree et al. 2007, Bull. Insect. 60, 151-152. | | |
| Stunting, little leaf | Mexico | | | | FJ951626 – FJ951629 | 16SrIII | | | Tapia-Tussell et al. 2012, Afr. J. Biotech. 11, 2169-2177. | | |
| Yellowing |  |  |  |  | - | 16SrI-B | | | Holguin-Pena and Vázquez-Juarez 2007, Pl. Dis. 91, 328. | | |
| Yellowing, stunting | Mexico | | | | KX092011 | 16SrVI | | | Salas-Munoz et al. 2016, Pl. Dis. 100, 2320-2320. | | |
| Yellowing | Oman | | | | - | 16SrII-D | | | Al-Subhi et al. 2018, Pl.Dis. 102, 576-588. | | |
| Stunt, phyllody | Poland | | | | - | 16SrI-C | | | Krawczyk et al. 2010, J.Phytopathol. 158, 496-502. | | |
| Witches’ broom | Romania and Russia | | | | HM449999 | 16SrXII-A | | | Ember et al. 2011, Eur. J. Pl.Pathol. 130, 367–377. | | |
| Stunt, malformation | Saudi Arabia | | | | KF017473-KF017475 | 16SrII | | | Alhudaib and Rezk, 2014, Int. J.Virol. 10, 180-191. | | |
| Stunt, malformation | Tanzania | | | | KR059873- KR059875 | 16SrII-C | | | Testen et al. 2015, Pl. Dis. 99, 1854. | | |
| Sepalhypertrophy, phyllody | Turkey | | | | JQ730750 | 16SrXII-A | | | Caglar et al. 2010, J. Turk. Phytopath. 39, 1-8. | | |
| Big bud | USA | | | | - | 16SrVI | | | Shaw et al. 1993, Pl. Dis. 77, 290-295. | | |
|  |  |  |  |  | - | 16SrI-A | | | Lee et al. 1993, Phytopathol.83, 834-842. | | |
| ***Solanummelongena* (brinjal, eggplant)** | | | | | | | | | | | |
| Little leaf | | Bangladesh | | AF228052 | | | 16SrVI-D | | | Siddique et al. 2001, J.Phytopathol. 149, 237-244. | |
|  |  |  |  | EU921446 | | | 16SrI | | | Kelly et al. 2009, Pl.Pathol. 58, 789. | |
| Giant calyx | | Brazil | | HM589212 HM589213 | | | 16SrIII-J, -U | | | Amaral-Mello et al. 2011, Int. J. Syst.Evol.Microbiol. 61, 1454-1461. | |
| Stunting, vascular necrosis | |  |  | HM237045 | | | 16SrIII-J | | | Rappussi et al. 2012, Eur. J. Pl.Pathol. 133, 829–840. | |
|  |  |  |  | JN818845 | | | 16SrXV-A | | | Canale and Bedendo 2013, Pl. Dis.97, 419–420. | |
| Stunting | |  |  | KR270802 | | | 16SrVII-B | | | Pereira et al. 2016, Pl.Dis.100, 1007. | |
| Phyllody | | China | | KC953000 | | | 16SrII-A | | | Cai et al. 2016, Ann. Appl. Biol. 169, 64-74. | |
| Phyllody, virescence | | Egypt | | FN257482 FR822706 | | | 16SrII-D | | | Omar and Foissac 2012, Eur. J. Pl. Pathol. 133, 353–360. | |
| Big bud | | India | | KT259050 | | | 16SrII-D | | | Yadav et al. 2016, Pl. Dis. 100, 517. | |
| Little leaf | |  |  | - | | | - | | | Varma et al. 1969; 1975, Ind.Phytopath.22, 289-291; Das and Mitra 2004, Ind.Phytopath.57, 242-244. | |
|  |  |  |  | JQ518317 | | | 16SrI | | | Kumar et al. 2012, New Dis. Rep.26, 21. | |
|  |  |  |  | EF186820 | | | 16SrVI-D  16SrVI-A | | | Martini et al. 2007, Int. J. Syst.Evol.Microbiol.57, 2037–2051. | |
|  |  |  |  | EU168777 | | |  |  |  | Hodgetts et al. 2008, Int. J. Syst.Evol.Microbiol. 58, 1826-1837. | |
|  |  |  |  | JQ409542 | | | 16SrVI-D | | | Azadvar and Baranwal 2012, Phytopath. Moll. 2, 15-21. | |
| Little leaf and stunting | |  |  | KP866409 KX689253  KX689254 | | | 16SrII-D | | | Kumar 2015, M.Sc Thesis, ICAR-IARI, New Delhi | |
| Little leaf, yellows | |  |  | KC178679 KP768069 KU509051  KU509052  KX689234- KX689252 | | | 16SrVI-D | | | Kumar et al. 2016, Virus Dis. 27, 207-208. | |
| Little leaf, yellows, witches’ broom, phyllody, stunt, big bud | |  |  | KP027530 | | | 16SrVI-D | | | Yadav et al*.* 2015, Int. J. Life Sci. 9, 109-112. | |
| Stunt, yellows, malformation | |  |  | JX464669 | | | 16SrIX-C | | | Tohidi et al. 2015, J.CropProt. 4, 247-256. | |
| Phyllody, little leaf | | Iran | | JX083377 | | | 16SrII-D | | | Siampour et al. 2012, Pl.Pathol. 62, 452-459. | |
| Big bud | |  |  | AB242231 | | | 16SrI-B | | | Okuda et al. 1997, Pl.Dis. 81, 301–305. | |
| Dwarfing | | Japan | | HQ423156 | | | 16SrII-D | | | Al-Subhi et al. 2011, Pl.Dis.95, 360. | |
| Phyllody, witches’ broom | | Oman | | HM450000 | | | 16SrXII-A | | | Ember et al. 2011, Eur. J. Pl.Pathol. 130, 367–377. | |
| Leaf yellowish discoloration | | Russia | | - | | | 16SrVI-A | | | Sertkaya et al. 2007, Bull. Insect. 60, 141-142. | |
| Yellowing | | Oman | | - | | | 16SrII-D | | | Al-Subhi et al. 2018, Pl.Dis. 102, 576-588. | |
| ***Solanumtuberosum*(potato)** | | | | | | | | | | | |
| Purple top | | Belgium | | - | | | 16SrXII-A | | | Tahzima et al. 2013, 65th ISCP, 649. | |
| Aerial tuber, hairy shoots | | Bolivia | | [AY725209](http://www.ncbi.nlm.nih.gov/entrez/query.fcgi?cmd=search&db=Nucleotide&dopt=GenBank&term=AY725209) | | | 16SrI | | | Jones et al. 2005, Pl.Pathol. 54, 234. | |
| Witches’ broom, yellow, stunting | | Canada | | - | | | 16SrVI; 16SrI | | | Khadhair et al. 2003, Acta Hort. 619, 167–176. | |
| Purple top | | China | | HQ609490 | | | 16SrVI-A | | | Cheng et al. 2015, Eur. J. Pl.Pathol.142, 305-318. | |
|  |  |  |  | HQ599228 | | | 16SrI-B | | |  |  |
|  |  |  |  | EU293841 | | | 16SrXII-E | | |  |  |
| Yellowing | | Colombia | | - | | | 16SrV | | | Mejia et al. 2011, Bull. Insect.64(Suppl.), S97-S98. | |
| Purple top | | Ecuador | | KT312845  KT312846 | | | 16SrII | | | Caicedo et al. 2015, New Dis. Rep. 32, 20. | |
| “Stolbur” | | Greece | | KJ810575 | | | 16SrXII-A | | | Holeva et al. 2014, Pl.Dis. 98, 1739. | |
| Stunting severe leaf mottling | | Indonesia | | EU273881 | | | 16SrII | | | Harling et al. 2009, Pl.Pathol.58, 791-791. | |
| Purple top | | Iran | | EU661607 FJ427295 | | | 16SrXII-A  16SrVI-D | | | Sichani et al. 2014, J.Pl.Prot. Res. 54, 1-8. | |
|  |  |  |  | AJ964960 | | | 16SrXII-A  16SrVI-A | | | Hosseini et al. 2011, J.Phytopathol. 159, 241–246. | |
| Yellowing, stunting | | Korea | | AB076404 | | | 16SrVIII | | | Jung et al. 2003, J. Gen. Pl.Pathol. 69, 87-89. | |
| Asymptomatic | | Italy | | - | | | 16SrX | | | Paltrinieriand Bertaccini, 2007, Bull. Insect. 60, 379-380. | |
| Purple top | | Mexico | | - | | | 16SrI,16SrII 16SrXIII | | | Santos-Cervantes et al. 2010, Pl. Dis.94, 388-395. | |
| “Stolbur” | | Montenegro | | KU588188-93 | | | 16SrXII-A | | | Radonjic et al. 2016, Pl. Dis. 10, 1094. | |
| Purple top | | New Zealand | | - | | | 16SrXII-B | | | Liefting et al. 2009, Pl. Dis. 93, 969. | |
| Stunt, purple top | | Pakistan | | - | | | - | | | Nasir et al. 2007, Bull. Insect. 60, 377-378. | |
| Stunting | | Pakistan | | FJ178388 | | | 16SrI | | | Fahmeed et al. 2009, J.Phytopathol.157, 639-641. | |
| Purple top | | Romania and Russia | | HQ108386  HQ108387 | | | 16SrXII-A | | | Ember et al. 2011, Eur. J. Pl.Pathol. 130, 367–377. | |
|  |  | Russia | | KP864673 KP864663  EU333398 EU344884 | | | 16SrI-A,  16SrI-B  16SrIII-B  16SrVI-C  16SrXII | | | Girsova et al. 2016, Eur. J.Pl.Pathol. 145, 139–153. | |
| Purple top | | Serbia | | KC703017 KC703019 | | | 16SrXII-A | | | Mitrovic et al. 2016, Eur. J. Pl.Pathol. 144, 619–630. | |
|  |  | Turkey | | AF248959 | | | 16SrXII-A | | | Caglar et al. 2010, J. Turk.Phytopathol. 39, 1-8. | |
| Stunt, purple top | | USA | | DQ174114-DQ174123 | | | 16SrI-A | | | Secor et al. 2006, Pl. Dis. 90, 377. | |
| Purple top | |  |  | DQ174118 DQ174121 | | | 16SrXVIII-A 16SrXVIII-B | | | Lee et al. 2006, Pl. Dis. 90, 989–993. | |
|  |  |  |  | - | | | 16SrVI-A | | | Lee et al. 2004, Pl. Dis. 88, 429. | |
| ***Capsicum annuum* (pepper, chili)** | | | | | | | | | | | |
| Little leaf | | Bolivia | FJ207456 | | | | | 16SrIII | | | Arocha et al. 2010, Pl.Pathol. 56, 345. |
| Little leaf | | China | - | | | | | 16SrII-D | | | Jie et al. 2010, Sci.Agr.Sin. 43. 304–312. |
| Little leaf, witches’ broom | |  | JF734910 KC008607 | | | | | 16SrI-B | | | Li et al. 2013, Phytoparasitica 41, 429-434. |
| Purpleveinsyndrome | | Costa Rica | HQ225624-35 | | | | | 16SrXXXI | | | Lee et al. 2011, Int. J. Syst.Evol.Microbiol. 61, 2822–2826. |
| Little leaf | | Cuba | [DQ286947](http://www.ncbi.nlm.nih.gov/entrez/query.fcgi?cmd=search&db=Nucleotide&dopt=GenBank&term=DQ286947) | | | | | 16SrI | | | Arocha et al. 2007, Pl.Pathol. 56, 345. |
| Witches’ broom | | Egypt | EU232715 | | | | | 16SrII | | | **El-Banna** et al. 2007, Egypt.J.Virol. 4, 93-111. |
| Witches’ broom | | India | KY612251 | | | | | 16SrVI-D | | | **Rao et al. 2017,** New Dis. Rep. 35, 33. |
| Little leaf stunting | | India | DQ343288 | | | | | 16SrI | | | Khan andRaj 2006, Pl.Pathol. 55, 822. |
| Little leaf, axillary buds | |  | - | | | | | - | | | Singh and Singh 2000, Ind.Phytopath.53, 309-310. |
| Big bud, little leaf | | Iran | KR706443 KR706444 | | | | | 16SrII | | | Faghihi et al. 2016, New Dis. Rep.33, 15. |
| Big bud | | Mexico | KM095133  KM095132 | | | | | 16SrVI-A  16SrVI-J | | | Mauricio-Castillo et al. 2015, Rev.Fitop.Mex.38, 389-396. |
| Little leaf | | Mexico | DQ092321 | | | | | 16SrI | | | Santos-Cervantes et al. 2008, Pl. Dis. 92, 1007-1011. |
| Witches’ broom | | Romania and Russia | - | | | | | 16SrXII-A | | | Ember et al. 2011, Eur. J. Pl.Pathol. 130, 367–377. |
| Yellows, stunting | | Czech Republic | - | | | | | 16SrXII | | | Navratil et al. 2009, Crop Prot. 28, 898-904. |
| Yellows, stunting | | Azerbaijan | - | | | | | 16SrXII | | | Balakishiyeva et al.2010, J. Pl.Pathol.92, S115. |
| Yellowing | | USA | Electron microscopy | | | | | - | | | Lebsky and Poghosyan 2007, Bull. Insect.60, 131-132. |
| Leaf mottling | | Indonesia | EU273881 | | | | | 16SrII | | | Harling et al. 2009, Pl.Pathol.58, 791. |
| Phyllody | | India | - | | | | | 16SrII-D | | | Sharma et al. 2015, Pl.Pathol.97, 548. |
| Phyllody | | Australia | - | | | | | 16SrII | | | Tran-Nguyen et al. 2003, Aus. Pl.Pathol.32, 559-560. |
| Short internodes, virescence | | Spain | - | | | | | 16SrVI | | | Castro and Romero 2002, J.Phytopath.150, 25–29. |
| “Brotegrande” | | USA | HQ436488 | | | | | 16SrVI | | | Randall et al. 2010, Pl. Dis.93, 968. |
| Green calyx | |  | FJ525437 | | | | | 16SrVI-A | | | Randall et al. 2009, Pl.Health Progress |
| Yellowing | |  | - | | | | | 16SrI-B | | | Lee et al. 1992,Phytopathol. 82, 977-986. |

| **Leguminaceae**  ***Pisumsativum* (Pea)** | | | | | | |
| --- | --- | --- | --- | --- | --- | --- |
| Stunting and proliferation | | Poland | | GU060495 | 16SrXII-A | Zwolinska et al. 2012,J.Phytopath. 160, 317–323. |
| **Viciafaba (Faba bean)** | | | | | | |
| Phyllody | | Saudi Arabia | | LN898424 LN898426 | 16SrII-D | Omar 2017,Eur. J. Pl.Pathol. 133, 353–360. |
| Yellowing and stunting | | Cuba | | [DQ286953](http://www.ncbi.nlm.nih.gov/entrez/query.fcgi?cmd=search&db=Nucleotide&dopt=GenBank&term=DQ286953) | 16SrI | Arocha et al. 2007, Pl.Pathol. 56, 345. |
| Phyllody | | Iran | | KP869128 KP869129 | 16SrII-C 16SrII-D | Salehi et al*.* 2016, Crop Prot. 89, 12-20. |
| Phyllody and witches’ broom | | Egypt | | - | - | Hamed et al. 2014,Int. J.Virol. 10, 129-135. |
| Phyllody and stunting | | Saudi Arabia | | JQ861532 JQ861533 | 16SrII | Al-Saleh and Amer2014, J. Anim. Pl. Sci. 24, 221-228. |
| Phyllody and witches’ broom | | India | | - | - | Singh et al. 2013, J. Environ. Biol. 34, 837-840. |
| Yellowing and stunting | | Sudan | | - | 16SrII-D | Alfaro-Fernandez et al. 2012,Eur. J.Pl.Pathol. 133, 791-795. |
| Phyllody | | Spain | | AJ557264 | 16SrIII | Castro and Romero 2004,J. Agric. Res. 2, 253-256. |
| Stunting and yellowing | | Lithuania | | - | 16SrIII-B | Valiunas et al. 2000, Proc. Development of environmentally friendly plant protection in the Baltic region, 28-29. |
| Phyllody | | Egypt and Sudan | | - | - | Dafalla and Cousin 1988, Agronomie 8, 441-449. |
| Witches’ broom | | Sudan | | - | - | Jones et al.1984, Pl.Pathol. 33, 599-602. |
| ***Lablab purpureus* (Garden bean)** | | | | | | |
| Yellowing | | Serbia | KM977907 | | 16SrXII-A | Mitrovic et al. 2015,Pl. Dis. 99, 551. |
| Yellowing, stunting | | Cuba | JN383914 | | 16SrI | Zamora et al. 2012,New Dis.Rep. 25, 4. |
| Big bud and little leaf | | India | LT558769 | | 16SrII-C | Thorat et al. 2016, Pl. Dis. 100, 252. |
| Interveinalchlorosis | | Costa Rica | AY496003 | | 16SrI | Moreira et al. 2009,New Dis. Rep. 19, 31. |
| Bud proliferation | | India | KP899065 | | 16SrII | Naik et al. 2015, New Dis.Rep. 31, 31. |
| *Canavaliaensifrmis* (Jack bean) | | | | | | |
| Yellowing, mosaic | Cuba | | KR232799 | | 16SrI-B | Acosta et al. 2015, J.Pl.Pathol. 97, 339-344. |
| **Vignaunguiculata (Cowpea)** | | | | | | |
| Witches’ broom | Australia | | - | | 16SrII-D | Saquib et al. 2006, Aus. Pl.Pathol. 35, 293-296. |
| Witches’ broom | India | | LT558768 | | 16SrII-D | Thorat et al. 2016,Pl. Dis. 100, 252. |
| Bud proliferation | India | | HM449952 | | 16SrI-B | Kumar et al. 2012,New Dis. Rep. 25, 28. |
| Little leaf | Australia | | - | | 16SrV | De La Rue et al. 2001,J.Phytopath. 149, 613–619. |
| Little leaf | India | | - | | 16SrXIV | Mall et al. 2015,Ind.Phytopath. 68, 449–453. |
| Flat stem | India | | KY439870 | | 16SrXIV-A | Rao et al. 2017,New Dis. Rep. 35, 33. |
| Lettuce | | | | | | |
| Yellowing and leaf distortion | USA | | AY38981 AY389822 AY38982 AY389828 | | 16SrI-B | Zhang et al. 2004, Phytopathol. 94, 842- 849. |
| Yellowing | USA | | KF573449-KF573456 | | 16SrI-A 16SrI-B | Lee et al. 2014,New Dis. Rep. 29, 5. |
| Yellowing |  |  |  |  |  |  |
| Yellowing | Italy | | - | | 16Sr1-B | Vibio et al. 1994,Phytopath. Medit. 33, 179-186. |
| Yellowing | USA | | - | | 16Sr1-A | Lee et al. 1993,Pl. Dis. 77, 815-817. |
| Yellowing | USA | | - | | 16SrI-B | Lee et al. 1992,Phytopathol. 82, 977-986. |
| Yellowing | Italy | | - | | 16SrI-B | Vibioet al. 1994,Phytopath. Medit. 33, 179-186; Marcone et al. 1997,J.Pl.Pathol. 79, 211–217. |
| Yellowing | Spain | | - | | 16SrI-B | Alfaro-Fernandez et al. 2011, Bull.Insect. 64(Suppl), S63-S64. |
| Yellowing | India | | EU362630 | | - | Arocha et al. 2008,Pl.Pathol. 17, 36. |
| Sprout proliferation and leaf deformation | Chile | | MG720234 | | 16SrIII-J | Quiroga et al. 2017, Phytopath. Moll. 7, 91-94. |
| Phyllodyvirescence witches’ broom | Iran | | AF515638 | | 16SrIX | Salehi et al. 2007,Pl.Pathol. 56, 669–676. |
| Carrot | | | | | | |
| Yellowing | Canada | | - | | - | Wally et al. 2004,Can. J. Pl.Pathol. 26, 498-505. |
| Leaf yellowing and reddening | Israel | | - | | - | Orenstein et al. 1999, J. Pl.Pathol. 81, 193–199. |
| Yellowing, malformation | Hungary | | - | | 16SrXII-A | Viczian et al. 1998, ActaPhytopath.Entomol. Hung. 33, 255-260. |
| Yellowing | USA | | - | | 16SrI-B | Lee et al. 1992,Phytopathol. 82, 977-986; Lee et al. 1993,Phytopathol. 83, 834-842. |
| Proliferation | Germany | | - | | 16SrI-B | Schneider et al. 1997,Microbiol. 143, 3381-3389. |
| Fasciation, phyllody, hairy roots | Saudi Arabia | | LN898420-LN898423 | | 16SrII-D | Omar 2017,J. Pl. Interact. 12, 58–66. |
| White leaf | United Kingdom | | EU362628 | | 16SrII-C | Arocha et al. 2008, Pl.Pathol. 17, 36. |
| Yellowing and reddening | United Kingdom | | KJ819956 | | 16SrI | Nisbet et al. 2014, New Dis. Rep. 30, 16. |
| Onion | | | | | | |
| Witches′ broom and phyllody | Mauritius | | GU129974 | | 16SrI-B 16SrXII–A | Gungoosingh-Bunwaree et al. 2010Pl. Health Progress |
| Yellowing, virescence | Japan, Italy | | - | | 16SrI-B | Nambaet al. 1993,Phytopathol.83781–786; Vibio et al. 1995,L’Inf.tore Agr. 35, 75-77 |
| Purple leaves | Saudi Arabia | | LN898434- LN898436 | | 16SrII-D | Omar 2017,J. Pl. Interact. 12, 58–66. |
| Leaf yellows | India | | KX641075 | | 16SrXI | Goel et al. 2017, Ind. Phytopath. 70, 368. |
| Yellows-type | Canada | | - | | 16SrI-A | Khadhair et al. 2002, Acta Hort. 619, 167–176. |
| Garlic | | | | | | |
| Yellows | Canada | | - | | 16Sr1-A | Khadhair et al. 2002,Acta Hort. 619, 167–176. |
| Leaf yellows | India | | KX641076 | | 16SrXI | Goel et al. 2017, Ind. Phytopath. 70, 368. |
| Okra | | | | | | |
| Stunting and yellowing | Mauritius | | - | | 16SrXII 16SrV | Gungoosingh-Bunwaree et al. 2011,Bull. Insect. 64(Suppl), S103-S104. |
| Bunchy top | India | | HQ828108 | | 16SrI | Kumar et al. 2012,New Dis. Rep. 25, 28. |
| Amaranthus | | | | | | |
| Yellowing | India | | EU362627 | | 16SrII | Ghosh et al. 1999, New Dis. Rep. 83, 302. |
| Yellowing | India | | - | | 16SrII | Arocha et al. 2008,Pl.Pathol. 17, 36. |
| Mosaics and bud proliferation | Mexico | | FJ357164 FJ357167 FJ390054 FJ390055 | | 16SrII | Ochoa-Sanchez et al. 2009, Phytoparasitica 37, 381-384. |
| Spinach | | | | | | |
| Yellows | Iran | | - | | 16SrI | Tazehkand et al. 2010, Ir. J.Phytopath. 158,713-720. |
| Celery | | | | | | |
| Leaf stunting and reddening | Australia | | - | | 16SrII-E | Tran-Nguyen et al. 2003, Aus. Pl.Pathol. 32, 559–560. |
| Severe yellowing | Italy | | - | | 16SrXII-A | Carraro et al. 2008,J. Pl.Pathol. 90, 131-135. |
| Leaf reddish discoloration | Serbia | | - | | 16SrXII-A | Ivanovic et al. 2011, Bull. Insect. 64(Suppl), S239-S240. |
| Stunting | Czech Republic | | - | | 16SrI-C | Franova and Spak 2013, J.Phytopath. 161, 666–670. |
| Yellowing | Spain | | - | | 16SrXII-A | Llacer and Avinent 1995, Plagas 21, 417-423. |
| Yellowing | Spain | | - | | 16SrXII-A 16SrI-B | Alfaro-Fernandez et al. 2011,Bull. Insect. 64(Suppl.), S63-S64. |
| Yellowing | Spain | | - | | 16SrXII-A | Murolo et al. 2010, J.Appl.Microbiol. 109, 2049-2059. |
| Yellowing | USA | | - | | 16SrI-B | Keane et al. 1996.Diagnostic in Crop Product 65, 263-268 |
| Leaf reddening | Romania | |  | | 16SrXII-A | Chireceanu et al. 2016, Phytopath. Moll. 6,46-49. |
